# Effect of Dexamethasone on the Expression of the α2,3 and α2,6 Sialic Acids in Epithelial Cell Lines

**DOI:** 10.3390/pathogens11121518

**Published:** 2022-12-12

**Authors:** Onasis Vicente-Fermín, Edgar Zenteno, Ivan Ramos-Martínez, Clara Espitia, José Ivan Sánchez-Betancourt, Leonor Huerta

**Affiliations:** 1 Departamento de Inmunología, Instituto de Investigaciones Biomédicas, Universidad Nacional Autónoma de México, Coyoacán, Ciudad de México 04510, Mexico; 2 Departamento de Bioquímica, Facultad de Medicina, Universidad Nacional Autónoma de México, Coyoacán, Ciudad de México 04510, Mexico; 3 Departamento de Medicina y Zootecnia de Cerdos, Facultad de Medicina Veterinaria y Zootecnia, Universidad Nacional Autónoma de México, Coyoacán, Ciudad de México 04510, Mexico

**Keywords:** sialic acid, A549, MDCK, Vero, dexamethasone, influenza receptors, zoonosis, sialylation

## Abstract

*N*-acetylneuraminic acid linked to galactose by α2,6 and α2,3 linkages (Siaα2,6 and Siaα2,3) is expressed on glycoconjugates of animal tissues, where it performs multiple biological functions. In addition, these types of sialic acid residues are the main targets for the binding and entry of influenza viruses. Here we used fluorochrome-conjugated *Sambuccus nigra*, *Maackia amurensis*, and peanut lectins for the simultaneous detection of Siaα2,3 and Siaα2,6 and galactosyl residues by two-color flow cytometry on A549 cells, a human pneumocyte cell line used for in vitro studies of the infection by influenza viruses, as well as on Vero and MDCK cell lines. The dexamethasone (DEX) glucocorticoid (GC), a widely used anti-inflammatory compound, completely abrogated the expression of Siaα2,3 in A549 cells and decreased its expression in Vero and MDCK cells; in contrast, the expression of Siaα2,6 was increased in the three cell lines. These observations indicate that DEX can be used for the study of the mechanism of sialylation of cell membrane molecules. Importantly, DEX may change the tropism of avian and human/pig influenza viruses and other infectious agents to animal and human epithelial cells.

## 1. Introduction

Terminal *N*-acetylneuraminic acid (Neu5Ac, Sia) linked to β-galactose by α2,3 or α2,6 linkages (Siaα2,3 and Siaα2,6, respectively) and mucin-type *O*-glycans containing Gal and GalNAc (Gal-β(1-3)-GalNAc, Thomsen–Friedenreich antigen) are present on glycoproteins and glycolipids on cell membranes and are involved in cell–cell interaction [1], signaling processes [2], cell–substrate interactions, and differentiation [3,4,5]. Particularly, aberrant expression of terminal α2,6 Neu5Ac and Gal-β(1-3)-GalNAc is found in a variety of tumors [6,7,8,9] The biological relevance of Siaα2,3 and Siaα2,6 is increased by the fact that they are the main receptors for influenza viruses that infect epithelial cells from several animal species and humans [10,11].

Dexamethasone (DEX) is a potent synthetic glucocorticoid derived from hydrocortisone, and so it regulates carbohydrate and protein metabolism. Pharmacologically, DEX has anti-inflammatory, anti-allergic, and immunosuppressive effects, so it is widely used in the treatment of respiratory distress and other inflammatory conditions both in humans and farm animals. Mechanisms of action of DEX are highly diverse and are mediated by binding to both cytoplasmic and membrane receptors, forming complexes (GC-GCR) which directly suppress the expression of genes encoding for pro-inflammatory proteins, inactivate transcription factors, and induce modification of gene expression by epigenetic mechanisms. Other effects described are the skewing of the cell metabolism by reduction of the intracellular ATP availability, promotion of the phagocytosis of apoptotic bodies, inhibition of the expression of adhesion molecules (thus inhibiting immune cell migration), and reduction of the production of prostaglandins reviewed in [12,13]. However, other effects of DEX are less studied. Particularly, there are several reports showing that it can modulate the expression of sialic acids on the cell surface in vitro [14] and increase the expression of sialyltransferases in fibroblasts [15]. Here, we report the effect of DEX on the expression of Siaα2,3 and Siaα2,6 on monkey and dog kidney epithelial cells and the A549 human pneumocyte cell line.

Binding of the fluorescent *Maackia amurensis* (MAAII) and *Sambucus nigra* (SNA) lectins is a widely used method for the detection of Siaα2,3- and Siaα2,6 on both primary tissues and cell lines. Most studies showing binding of these lectins are conducted using fluorescence microscopy [10,16,17]. Reports using these lectins in the analysis of the expression of terminal glycans by flow cytometry can be found in the literature, with mo, st using single lectin labeling [18,19,20,21,22,23]. The simultaneous detection of lectin binding on the cell surface would further demonstrate binding specificity and provide an abbreviated assay for the analysis of the concurrent expression of glycans.

Here, we used a two-color flow cytometry assay for the specific and simultaneous detection of the effect of DEX on the binding of SNA and MAAII to the surface of three cell lines widely used for influenza research. A549 is a human alveolar type II epithelial cell line (type II pneumocytes) [24] and is the most used model for the in vitro study of the infection of human low respiratory tract cells by influenza viruses, which, along with an aberrant innate immune response, greatly relates to pathogenesis [25,26]. On the other hand, MDCK and Vero cells are widely used for the study of the virus mechanisms of entry, as well as for its growing in vitro, as an alternative to the allantoic cavity of embryonated chicken eggs [27,28,29]. The specificity of the binding of MAAII and SNA was demonstrated by the treatment of cells with neuraminidase, which exposes the non-sialylated terminal galactose moiety of *O*-glycans, which were detected by the use of the peanut lectin (PNA). Our results show that DEX exert a differential effect on the expression of Siaα2,3- and Siaα2,6.

## 2. Material and Methods

### 2.1. Cell Lines

Low-passage Madin–Darby canine kidney cells (MDCK.2; ATCC, Rockville, MD, USA) were cultivated in Eagle’s Minimum Essential Medium (EMEM, ATCC) supplemented with 10% inactivated fetal calf serum (By Productos; Guadalajara, Jalisco, México). A549 cells (ATCC) were kept in Kaighn’s modification of Ham’s F-12 medium (F12K, ATCC) including 10% FCS. Vero cells were obtained from Dr. Bertha Espinoza at Instituto de Investigaciones Biomédicas, UNAM, and cultured in EMEM. All cells were maintained at 37 °C in a 5% CO_2_ atmosphere. For passing, cell monolayers were washed twice with PBS and detached by digestion with 2 mL of 0.25% trypsin (Gibco, Gaithersburg, MD, USA) for 10 min at 37 °C. Trypsin was then inactivated by addition of 2 mL of supplemented culture medium. Cells were transferred to 15 mL centrifuge tubes, washed with 2 mL of PBS, resuspended in their respective culture medium, and maintained at 37 °C with 5% CO_2_.

### 2.2. Lectins

Biotin-conjugated *Maackia amurensis* agglutinin II, FITC-conjugated *Sambucus nigra* agglutinin, FITC-conjugated peanut agglutinin, and biotin-conjugated peanut agglutinin were used for flow cytometry analysis. Biotin-conjugated lectins were detected by using PE-conjugated streptavidin. Table 1 indicates the lectins, conjugated fluorochromes, produced color signal, specificity, and provider of these reagents.

### 2.3. Dexamethasone Treatment

A549, Vero, and MDCK cells were collected from cultures in logarithmic growth phase by trypsin digestion. After trypsin inactivation and washing, cells were resuspended in PBS and distributed at 5 × 10^5^ cells/50 μL in 1.6 mL microtubes. Cells were treated with 20 μM Dexamethsone phosphate, or DEX (Alin; Chinoin, CDMX, México) in the respective culture medium without serum and incubated at 37 °C with 5% CO_2_. After 12 h, cells were washed with PBS, stained, and processed for flow cytometry for the analysis of sialic acid expression.

### 2.4. Flow Cytometric Analysis of Lectin Binding

Before addition to cells, pairs of lectins and PE-conjugated streptavidin (for detection of biotin-conjugated MAAII or PNA) were combined at 10 μg/mL each in PBS (lectin working solution). For inhibition of lectin binding to cells by galactose and fetuin (Sigma Aldrich, Saint Louis, MO, USA), lectin working solution was prepared in 100 mM galactose or 0.1 mM fetuin in PBS. Thirty-five microliters of lectin working solution was then added to cells and incubated for 60 min at 4 °C. Cells were washed once with 1 mL of PBS, resuspended in 0.5 mL of the same buffer, and analyzed immediately in an Attune™ flow cytometer (blue/red lasers) from Thermo Fisher Scientific (Carlsbad, CA, USA). For neuraminidase treatment, 30 μL of neuraminidase type V from *Clostridium perfringens* (Sigma Aldrich) stock solution (6.1 U/mL) was added to the corresponding cells and incubated for 1.5 h at 37 °C, 5% CO_2_. Cells were washed with 1 mL of PBS before staining.

Unlabeled cells, single-green and single-red labeled cells were used to set up voltage and compensation settings. Double-labeled cells were then analyzed under the same conditions. Regions of viable cells in FSC-H vs. SSC-H were depicted, and green and red fluorescence was determined.

### 2.5. Statistical Analysis

Geometric mean fluorescence intensity data were collected and differences between groups were analyzed for significance by ANOVA (*p* < 0.05) or Student’s *t*-test (*p* < 0.05) using GraphPad Prism version 9.0 (GraphPad Software Inc.; San Diego, CA, USA).

## 3. Results

### 3.1. Two-Color Flow Cytometry Analysis of the expression of Siaα2,3, Siaα2,6, and Galβ1–3GalNAc Glycans on A549 Cells

Table 1 indicates the conjugated fluorochrome, produced color signal, specificity, and provider of the lectins used in these analyses.

**Figure 1:**
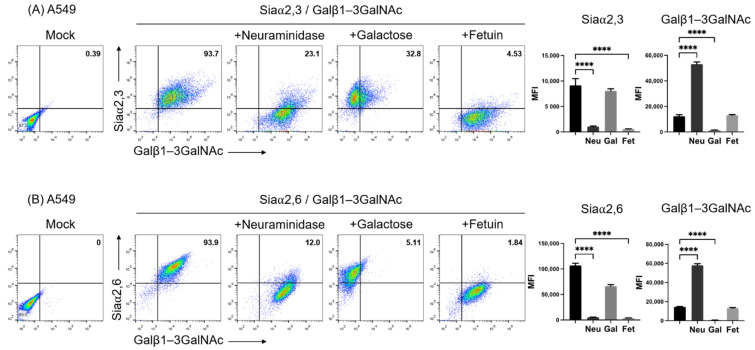
Two-color flow cytometry analysis of the expression of (**A**) Siaα2,3 and Galβ1-3GalNAc and (**B**) Siaα2,6 and Galβ1-3GalNAc on A549 cells by means of binding of SNA, MAAII, and PNA lectins. The effect of treatment of the cell surface with neuraminidase, blocking of PNA binding with galactose, and lectin absorption with fetuin is shown. The percentage of positive cells in the upper right quadrants is shown. The mean fluorescence intensity of triplicates in a representative experiment is shown on the right. MFI, mean fluorescence intensity; Gal, galactose; Neu, neuraminidase; Fet, fetuin. Significant differences are marked with asterisks (**** *p* < 0.0001, one-way ANOVA).

Removal of Siaα2,3 and Siaα2,6 from the cell surface by incubation with neuraminidase uncovers terminal galactosyl (β-1,3) *N*-acetylgalactosamine (Galβ1–3GalNAc) on glycoconjugates. Thus, neuraminidase treatment reduces SNA and MAAII, whereas it increases PNA binding [30]. Figure 1 shows the two-color flow cytometry analysis of the expression of Galβ1-3GalNAc and Siaα2,3 (Figure 1A) and Galβ1-3GalNAc and Siaα2,6 (Figure 1B), along with the effect of treatment of the cell surface with neuraminidase on A549 cells. Cells were clearly stained with the three lectins. As expected, neuraminidase greatly reduced the fluorescence of SNA and MAAII, whereas it increased that of PNA. As expected, galactose completely blocked the binding of PNA, whereas fetuin reduced the binding of both lectins to similar levels, since it absorbed lectins from the labeling solution. Thus, the two-color assay allowed a ready detection of Siaα2,3 and Siaα2,6 and the exposure of *O*-galactosyl residues by neuraminidase treatment.

### 3.2. Two-Color Flow Cytometry Analysis of the Expression of Siaα2,3 and Siaα2,6 Glycans on A549, Vero, and MDCK Cells

**Figure 2:**
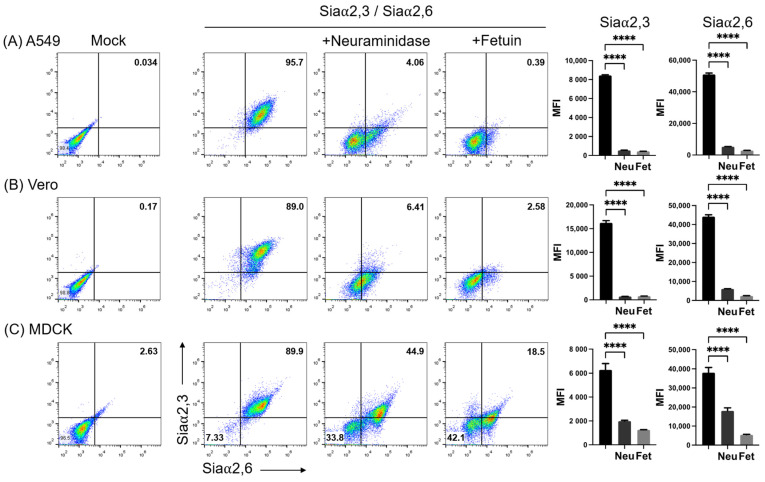
Effect of neuraminidase on the expression of Siaα2,3 and Siaα2,6 on (**A**) A549, (**B**) Vero, and (**C**) MDCK cells. The effect of treatment of the cell surface with neuraminidase and lectin absorption with fetuin is shown. The mean fluorescence intensity of triplicates in a representative experiment is shown on the right. FMI, mean fluorescence intensity; Neu, neuraminidase; Fet, fetuin. Significant differences are marked with asterisks (*p* < 0.0001, one-way ANOVA).

Figure 2 shows the simultaneous analysis of the expression of Siaα2,3 and Siaα2,6 on A549, Vero, and MDCK cells. As before, the neuraminidase treatment completely abrogated MAAII and greatly reduced SNA binding to A549 and Vero cells (third panels in Figure 2A,B). The remnant SNA fluorescence observed in a subpopulation after neuraminidase treatment (lower right quadrant) may be explained by the more efficient cleavage of Siaα2,3 than Siaα2,6 by *Clostridium perfringens* neuraminidase [31]. Instead, the adsorption of lectins by fetuin in the incubation medium completely inhibited binding in A549 and Vero cells (fourth panels in Figure 2A,B).

Simultaneous staining of MDCK.2 cells with SNA and MAAII revealed two cell populations with slightly different levels of expression of Siaα2,3 and Siaα2,6 (second panel in Figure 2C), which became clearly resolved after neuraminidase treatment (third panel of Figure 2C). Thus, the same concentration of neuraminidase that effectively removed lectin binding in A549 and Vero cells, completely abrogated SNA and MAAII fluorescence in only one subpopulation of MDCK cells, whereas another subpopulation was more resistant to the enzyme activity, with only a marginal reduction in total fluorescence for Siaα2,3. Heterogeneity of MDCK.2 cells was also indicated by the observation that fetuin, although inhibited binding of both lectins to a greater degree than neuraminidase, also generated two populations differing in the degree of inhibition of SNA fluorescence, with one of them being more resistant to fetuin competition for Siaα2,6 (fourth panel of Figure 2C).

Thus, double staining evidenced that MDCK.2 cells contained subpopulations harboring slightly different levels of expression of Siaα2,3 and Siaα2,6, which, however, showed marked different sensitivities to neuraminidase and fetuin activities.

### 3.3. Effect of Dexamethasone on the Expression of Siaα2,6 and Siaα2,3 Glycans on A549, Vero, and MDCK Cells

**Figure 3:**
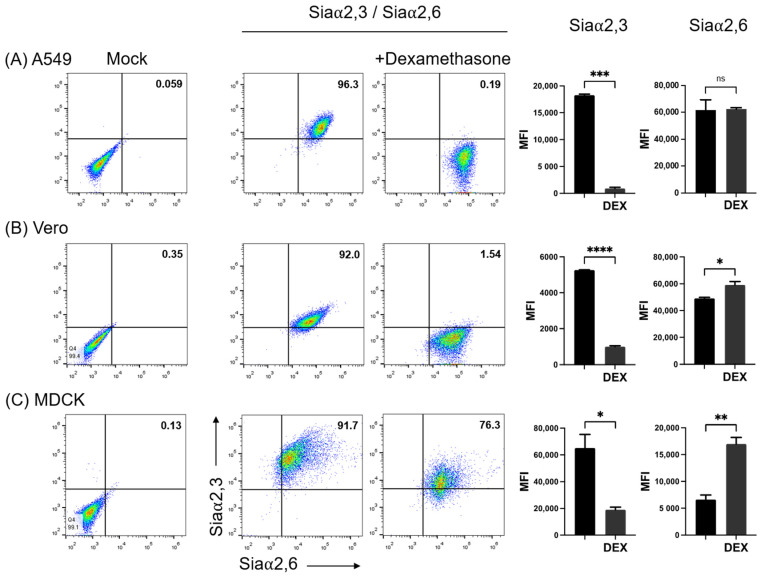
Effect of treatment of cells with dexamethasone (DEX)on the expression of Siaα2,3 and Siaα2,6 on (**A**) A549, (**B**) Vero, and (**C**) MDCK cells. FMI, mean fluorescence intensity; DEX, dexamethasone; ns, non-significative difference. Significant differences are marked with asterisks (*, *p* < 0.05, **, *p* < 0.01, ***, *p* < 0.001, ****, *p* < 0.0001; Student’s *t*-test).

**Figure 4:**
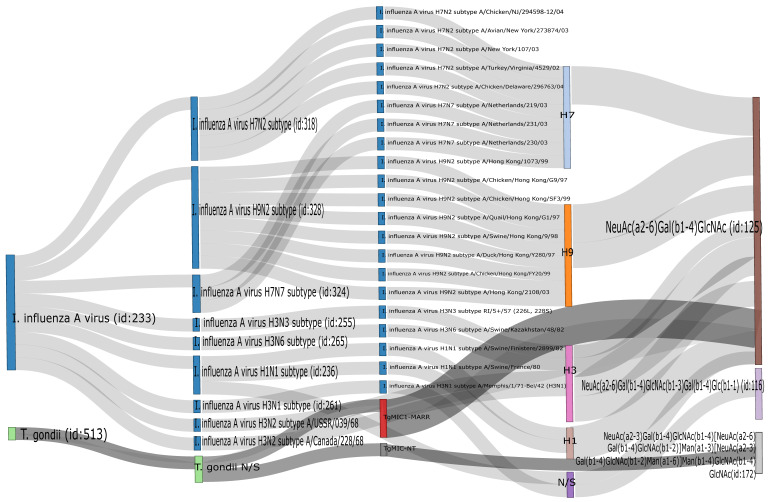
Output of the search in the SugarBind Database showing associations between pathogens and glycans. The query NeuAc(α2-6)Gal(β1-4)GlcNAc, the trisaccharide recognized by SNA, returned all pathogens matching these criteria. Influenza A H1, H3, H7, and H9 and *Toxoplasma gondii* recognize this glycan.

It has been previously shown that DEX decreases the expression of Siaα2,3 in Vero cells and that consequently the susceptibility of this cell line to infection with the porcine rubulavirus is greatly reduced [32]. We compared the effect of DEX on the expression of sialyl residues in A549, Vero, and MDCK.2 cells. DEX completely abolished the expression of Siaα2,3 in A549 cells and greatly reduced it in Vero and MDCK cells; in contrast, DEX increased the expression of Siaα2,6 in the three cell lines (Figure 3B). A request to the SugarBind Database from Expasy.org for the sequence of glycans recognized by SNA that were overexpressed by the effect of DEX was made. Several influenza A virus subtypes (H1, H3, H7, H9), as well as *Toxoplasma gondii*, an intracellular parasite, could potentially bind to glycans detected by SNA (Figure 4).

## 4. Discussion

Sialic acids are a family of monosaccharides comprising about 50 derivatives of neuraminic acid; they are widely distributed in nature as terminal sugars of oligosaccharides attached to proteins or lipids. Sialic acids are linked to galactose via α2,3 or α2,6-linkage or linked via α2,6-linkage to galactosamine or *N*-acetylgalactosamine [33]. Tools for the identification of Sia residues on cells and tissues comprise different methodologies such as histochemistry. Here we show that flow cytometry allows the simultaneous accurate analysis of the binding of SNA and MAAII lectins on three cell lines widely used for influenza studies. The concurrent exposure of additional *O*-galactosyl residues after neuraminidase treatment was also detected on the same cells along with reduced SNA or MAAII binding. Levels of fluorescence after treatment with neuraminidase and fetuin show that background fluorescence is minimal, confirming the specificity of the test.

It has been reported that dexamethasone modifies the expression of Siaα2,3 and increases that of Siaα2,6 in thymocytes, macrophages, and CHO cells [1,18,34]. An early report showed that dexamethasone decreases the expression of Siaα2,3 in Vero cells, limiting the infection of this cell line with the porcine rubulavirus [32]. By the simultaneous analysis of the two forms of sialic acid linkage, we confirmed that dexamethasone completely abolished the expression of Siaα2,3, whereas it slightly increased the expression of Siaα2,6 in Vero, MDCK, and A549 cells. Several studies have shown the correlation between the expression of Siaα2,3 and Siaα2,6 and the susceptibility to influenza viruses. Our observations show that treatment with dexamethasone may reduce the cell’s susceptibility to avian influenza viruses while maintaining or increasing their susceptibility to human and porcine influenza viruses.

The capacity of dexamethasone to abrogate the expression of Siaα2,3 but not of Siaα2,6 in A549 cells can be used in studies on the receptor specificity of influenza viruses and in the selective propagation of human viruses, which preferentially use Siaα2,6 for entry [11]. On the other hand, our observations show that dexamethasone can be used for the analysis of mechanisms controlling sialylation, which are scarcely investigated and may involve the regulation of the activity of particular sialyltransferases [35].

Further analysis of the specificity of the SNA lectin establishes that the minimal determinant it recognizes is Neu5Acα2-6Galβ1-4GlcNAc, the presence of this trisaccharide being necessary for binding [36]. The development of glycomics databases has provided a valuable tool to understand the interaction between pathogens and hosts through glycans, as is the case with the SugarBind database [37]. Searching for pathogens that could potentially recognize the trisaccharide Neu5Acα2-6Galβ1-4GlcNAc showed that hemagglutinin from several influenza A virus subtypes (H3, H7, and H9), as well as *Toxoplasma gondii,* could potentially bind to glycans detected by SNA.

The simultaneous determination of Siaα2,3 and Siaα2,6 along with *O*-galactosyl residues by flow cytometry can be applied to the analysis of influenza receptors as well as to cell–cell and cell–substrate interactions in other cell types. In addition, given its sensitivity and specificity, and restriction of detection of terminal glycan molecules at the cell surface, flow cytometry analysis can be used to detect glycan molecules that are expressed aberrantly on the surface of cancer cells, as a tool for diagnosis, prognosis, and therapy [7].

Two populations differing in sensitivity to *Clostridium perfringens* neuraminidase were observed in the MDCK.2 cell line. Heterogeneity of the original MDCK parental cells (CCL-34) was described before, showing that single-cell clones established from this cell line differed in their capacities to support replication of influenza A and B viruses and also in the requirement of trypsin for infection [23]. Cells used here specifically pertain to the MDCK.2 clone, which originates from CCL-34, and our analysis indicates that this clone may still contain significant heterogeneity. Awareness of the heterogeneity of MDCK cell lines is important for the interpretation of results obtained in influenza and toxoplasmosis research.

## 5. Conclusions

In summary, we demonstrated that dexamethasone treatment greatly reduces the expression of Siaα2,3 and conversely increases the expression of Siaα2,6 in animal and human epithelial cell lines; however, the mechanisms by which it regulates sialylation are scarcely investigated although may involve the regulation of the activity of specific sialyltransferases. Further studies are required to analyze its mechanisms of action. The present study suggests that dexamethasone may change the susceptibility of epithelial cells to zoonotic diseases such as influenza and toxoplasmosis. These observations support the need for in vivo studies on the effect of dexamethasone on sialic acid expression, given its potential implications in the susceptibility to infectious diseases.

## Figures and Tables

**Table 1:** Lectins used in this study.

Lectins	Conjugated Fluorochrome	Color Signal	Sialydated Residue	Manufacturer
Sambucus nigra	FITC	Green	Siaα2,6	Vector
Maackia amurensis II	Biotin/PE-streptavidin	Red	Siaα2,3	Vector
Peanut agglutinin	Biotin/PE-streptavidin	Red	Galβ1-3GalNAc	Vector
Peanut agglutinin	FITC	Green	Galβ1-3GalNAc	Sigma-Aldrich
